# Urine microbial fuel cells in a semi-controlled environment for onsite urine pre-treatment and electricity production

**DOI:** 10.1016/j.jpowsour.2018.08.051

**Published:** 2018-10-01

**Authors:** Clement A. Cid, Andrew Stinchcombe, Ioannis Ieropoulos, Michael R. Hoffmann

**Affiliations:** a Linde+Robinson Laboratories, California Institute of Technology, Pasadena, CA, USA; b Bristol BioEnergy Centre, Bristol Robotics Laboratory, University of the West of England, BS16 1QY, UK

**Keywords:** Microbial fuel cell, Electricity generation, Urine treatment

## Abstract

Microbial fuel cell (MFC) systems have the ability to oxidize organic matter and transfer electrons to an external circuit as electricity at voltage levels of <1 V. Urine has been shown to be an excellent feedstock for various MFC systems, particularly MFCs inoculated with activated sludge and with a terracotta ceramic membrane separating carbon-based electrodes. In this article, we studied a MFC system composed of two stacks of 32 individual cells each sharing the same anolyte. By combining the current produced by the 32 cells connected in parallel and by adding the potential of both stacks connected in series, an average power density of 23 mW m^−2^ was produced at an effective current density of 65 mA m^−2^ for more than 120 days. [NH_3_], TIC, COD, and TOC levels were monitored frequently to understand the chemical energy conversion to electricity as well as to determine the best electrical configuration of the stacks. Archaeal and bacterial populations on selected anode felts and in the anolyte of both stacks were investigated as well. Indicator microorganisms for bacterial waterborne diseases were measured in anolyte and catholyte compartments to evaluate the risk of reusing the catholyte in a non-regulated environment.

## Highlights

• Continuous averaged power density of 23 mW m^−2^ was produced for more than 120 days.
• COD and TOC removal was observed concomitantly with power production via anodic oxidation.
• Bacterial cross-over inside MFC was low when electrically connected in series or parallel.
• A large diversity of microorganisms was observed on the anodes and in solution.

## 1 Introduction

Energy recovery from waste is a major challenge at a time in which the Earth's resources are increasingly strained by human exploitation [1]. For instance, a 2012 special issue of *Science* focused on “Working with Waste” to minimize the use of raw materials [2]. One attractive way to recover part of the estimated 1.5 ⋅ 10^11^ kWh of chemical and physical energy lost from the wastewater rejected annually in the United States, is through the use of respiration of microbes in microbial electrochemical technologies [3] such as microbial fuel cells (MFCs). However, efficiently recovering useful amounts of energy from sewage at large scale treatment plants, is —at present— a suboptimal process because the nutrients containing most of the chemical energy of the wastewater have been highly diluted in the sewers [4]. The key is then to recover the chemical energy close to the source (*e.g.*, the toilet) before dilution. Urine-diversion toilets, with urine collection systems, have been employed in certain parts of the World, but even though urine is pathogen-free for healthy individuals, its potential contamination with fecal material [5] and its high ammonia and mineral content often prevent it from safe and user-friendly nutrient recovery in peri-urban and urban communities [6]. It has previously been reported that urine can successfully be used as a direct feedstock for certain microbes [7] that will oxidize some of its nutrients and transfer electrons to an inert substrate via direct or indirect processes as the anodic part of a MFC system [8,9]. This direct energy recovery and conversion to electricity from urine has shown promising results in standalone MFC systems [7,10] with a high power production per biomass for terracotta ceramic MFCs [11]. Such systems can also be installed in an onsite self-contained human waste treatment system relying on electrolysis to remove nitrogen, chemical oxygen demand (COD), pathogens, and to recover phosphorus [12]. MFC systems can also be used as a pre-treatment for COD and Total Organic Carbon (TOC) removal of urine coming from waterless urinals (Fig. S1). In this article, we investigate the operation of a MFC system for the pre-treatment of human urine by anodic microorganisms with electrical energy recovery. While this usage of MFC can lower the energy cost for treating human waste, it can also recover electrical energy in order to divert the urine flow, making this approach an overall energy gain for the entire onsite self-contained human waste treatment system.

## 2 Materials and methods

### 2.1 MFC stacks

The two versions of a similar design of MFC stacks employed in this study were installed in a public restroom on the campus of the California Institute of Technology (Caltech) in Pasadena (California, USA). The differences in the design are highlighted when necessary. Version A was used for the bacterial cross-over and current efficiency characterizations. Version B was used for long term monitoring with electrical energy harvesting.

**Fig. 1:**
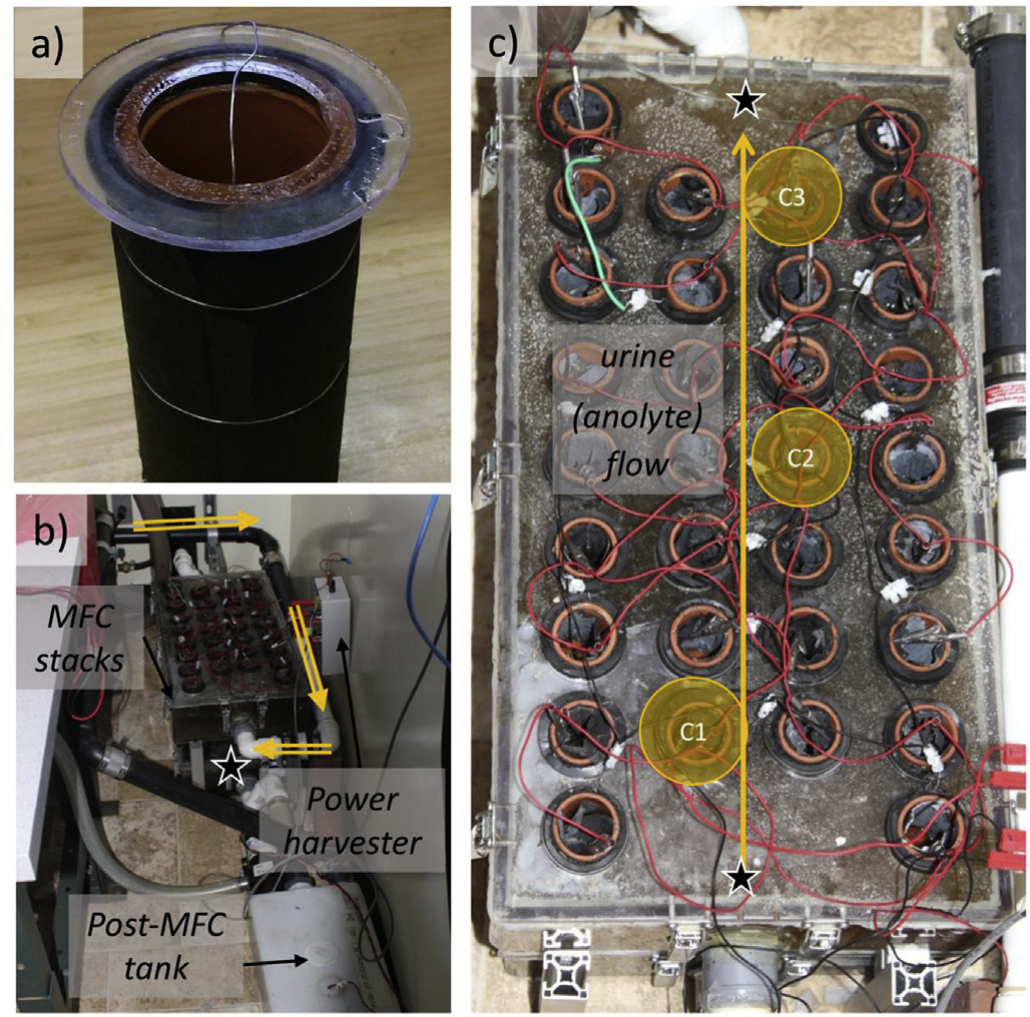
a) Picture of an empty terracotta microbial fuel cell with the anode supported by a nickel-chromium wire. b) Two MFC stacks on top of each other fed by gravity (the output of the top stack is connected to the input of the bottom stack at the outermost parts of the rectangular tank) and installed behind a water-free urinal on Caltech campus. c) Top view of the top MFC stack (version A) with direction of the gravity-fed urine flow through the system. Cells C1, C2, and C3 used for catholyte sampling for microbial testing (Table 1) are highlighted. Sampling points for the anolyte in top and bottom stacks are marked with a star.

**Fig. 2:**
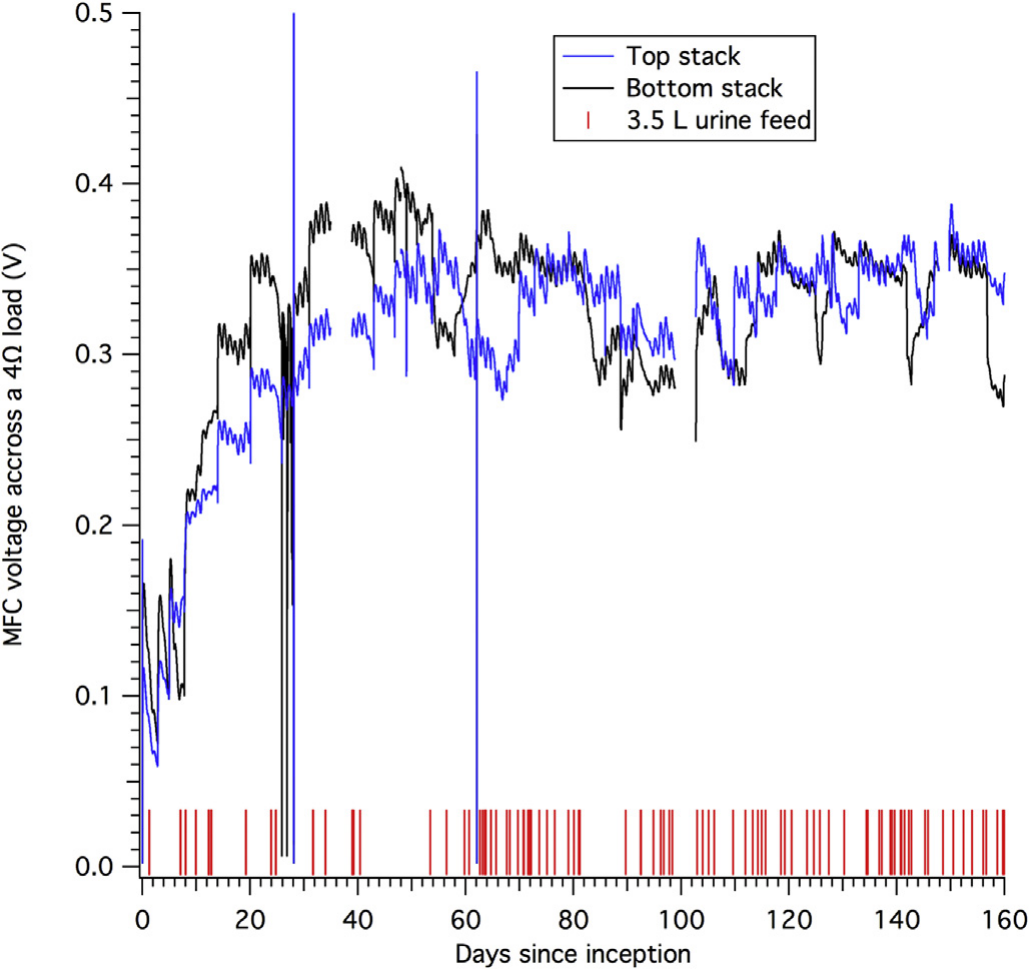
Voltage across the bottom and top stack (version B), each connected to a separate 4Ω individual load. Recorded urine feeding events are represented with vertical red bars. (For interpretation of the references to color in this figure legend, the reader is referred to the Web version of this article.)

Two MFC stacks for each version consisted each of 32 individual cells per stack (Fig. 1a) separated evenly and suspended in a rectangular tank connected to a water-free urinal (Fig. 1b). A gravity-driven cross-flow through each stack was made possible by placing the input and output connection at the outermost parts of the rectangular tank (Fig. 1c). In normal operation, the input of the top stack was connected to an equalization tank equipped with a level sensor commanding a pump. About 3.5 L of the urine drained from the water-free urinal were pumped when the level of urine in the equalization tank reached a certain height. The residence time of urine in the equalization tank could vary from few hours to few days as shown by the recorded feeding intervals in Fig. 2. The output of the top stack was connected to the input of the bottom stack with two 90° bent pipes to minimize cross-over between the top and the bottom stack. The output of the bottom stack drained by gravity into a tank for further processing.

The cells in both versions A and B were similar to the ones described by Salar-García *et al.* [10]: each cell had a terracotta tubular ceramic tube of 150 mm length and 42 mm internal diameter (50 mm outer diameter) with an unknown pore size (Weston Mill Pottery, Newark, UK) open to air in its center and acting as an ion-conductive separator between anode and cathode. Each anode was 1000 mm by 260 mm carbon veil (loading 20 g m^−2^, PRF Composite Materials, Dorset, UK) folded in half along its length to make 1000 mm by 130 mm and wrapped around the outside surface of the terracotta tubular ceramic tube. This was held in place by a stainless-steel wire. The wire was physically holding the electrode against the terracotta tube and acted as a current collector connected directly to the other anodes via alligator clips and metal wires (version A) or through an electrical bus bar attached to the stack acting as the anodic current collector (version B). The cathode was a 140 mm by 130 mm carbon veil with micro pores described elsewhere [13]. The cloth was rolled along its length (140 mm) and placed inside the terracotta tube in a manner intended to maximize the contact with the ceramic wall while reaching the bottom of the tube. Alligator clips connected to each other (version A) or to a metal bus bar cathodic current collector (version B) were used for electrical contact with the cathode cloth.

The inoculation period was similar for version A and version B and lasted approximately 24 days. Each stack was first inoculated with 10 L of a 1:1 solution of human urine and activated sludge from a local domestic wastewater treatment plant treating mostly domestic wastewater (San José Creek Water Reclamation Plant, Whittier, California, USA) for 3 days. After the initial addition, 3–6 L of urine were added to each stack at regular intervals (Fig. S2). The stacks were drained of the same volume before urine addition. During the inoculation, the anodes and cathodes were connected to a 4 Ω load, and voltage across the load was recorded on a continuous basis via an automatic data logger, *vide infra*. The inoculation period was stopped when the voltage across the resistor stabilized (not taking into account diurnal variations) (Fig. S2).

### 2.2 Electronics for performance monitoring and energy harvesting

The wires (version A) or metal bus bar (version B) from each stack were connected directly to a power harvester. The power harvester did not contain any active electrical components but facilitated connecting the stacks in series or parallel. The electrical energy of each stack was dissipated by the ‘Joule effect’ through an adjustable load with a potentiometer of 1–25 Ω range with 0.2 Ω precision (Digikey, USA). The electrical potential across the load was measured and recorded every 10 s by a two-channel data logger (Programmed Scientific Instruments, Arcadia, California, USA) connected to a Panel PC PPC-L62T (Advantech, China) with a dedicated software package (Program Scientific Instruments, Arcadia, California, USA). The potentiometers and the data logger electrical connections were adjusted to fit an independent, series, or parallel wiring between the two stacks.

### 2.3 Solution sampling and chemical analyses

Grab samples were taken from approximately 10 cm below the surface of each stack through a hole drilled as close to the inlet/outlet as possible. A 50-mL plastic syringe (BD, Franklin Lakes, New Jersey, USA) connected to a 15-cm piece of Tygon tube (Saint-Gobain, France) was used to collect between 10 mL and 20 mL of the solution. The syringe and the tube were rinsed with ultrapure water and dried multiple times with several suction/injection movements between each sampling. After the last sample was taken, the syringe and the tube were cleaned with a 10% solution of bleach and rinsed several times with ultrapure water and then allowed to dry until the next sampling.

Sampled solutions were filtered through a 25-mm Acrodisc Syringe Filters with a 0.45-μm GHP membrane (Pall Corporation, Port Washington, New York, USA) and diluted appropriately with ultrapure water before storage at 4 °C and analysis.

COD was measured in duplicates and triplicates via the colorimetric method Hach 8000 (Hach Company, Loveland, Colorado, USA). Ammonia [NH_3_] (measured as [NH_4_^+^]) was determined by ion chromatography (Dionex ICS 2000; AS19G anions, CS12A cations). TOC and Total Inorganic Carbon (TIC) concentrations were measured with an Aurora 1030W TOC Analyzer (OI Analytical, College Station, Texas, USA) using the heated persulfate wet oxidation technique.

### 2.4 Coulombic efficiency

Coulombic efficiency *ε* for COD removal (%) was determined using equation (1) proposed by Logan *et al.* [14] with the approximation that the MFC stacks were receiving an average daily flow *q* = 1 L day^−1^. Other parameters were *M* the molar mass of oxygen (*M* = 32 g mol^−1^), ℱ the Faraday's constant (ℱ  = 96,500 C mol^−1^), and *b* the number of electrons exchanged per mole of oxygen reduced (*b* = 4). *I* (A) was the averaged current going through the MFC stack as determined by Ohm's law (*E* = *RI*, *E* being the potential (V) across the resistor *R* (Ω) and *I* the current flowing through R). $\Delta_{\text{COD}}$ (mg O_2_ L^−1^) was the difference in COD values between influent and effluent.

(1) $$\varepsilon =\frac{MI}{ℱbq\Delta_{\text{COD}}}\cdot 100$$

### 2.5 Analyses of biomass in suspension

Three catholyte compartments from the top stack (C1, C2, and C3, Fig. 1c) were sampled as well as inside the anolyte compartment of the top and the bottom stacks. The sampling of the anolyte was performed close to the inlet and the outlet of the top stack and close to the outlet of the bottom stack. The samples were taken with a similar apparatus previously described.

Bacterial cross-over between anolyte and catholyte was assessed by estimating the quantity of four indicator organisms in Colony-Forming Units per mL (CFU mL^−1^) *Escherichia Coli (E. Coli)*, *Fecal Coliform*, *Total Coliform*, and *Enterococcus* with the following respective EPA methods: 1103.1 [15], Microbiological Method III.C-2 [16], 9132 [17], and 1600 [18] with appropriate dilutions.

### 2.6 Biological analyses of the anodes

Bacterial population estimates on the sampled carbon veils that were used as anodes for 4.5 months were determined by staining a small portion of a carbon veil anode using a LIVE/DEAD BacLight Bacterial Viability Kit L7012 (Molecular Probes, Eugene, Oregon, USA). The kit contained an appropriate mixture of SYTO 9 (excitation at about 480 nm, emission in green at about 500 nm) and propidium iodide (excitation at about 490 nm, emission in red at about 635 nm). The carbon veil was infused with enough volume of the dye mixture to cover all the veil sample following the manufacturer's recommendations. The dyed veil was then placed on a glass slide and observed under a fluorescent light microscope (Leica DMi 8, Leica Microsystems, Wetzlar, Germany) controlled by LAS X Expert software and equipped with the following fluorescence filter cubes: DAPI (blue color), FITC (green color), and RHOD (red color).

16S rRNA metagenomic sequencing was performed on selected anode and anolyte samples based on the analytical method developed by Kozich *et al.* [19] DNA was extracted using a Mo Bio PowerWater DNA isolation kit (QIAGEN, Germantown, Maryland, USA) [31] following a modified extraction method described in the Supplementary Information section.

## 3 Results and discussion

### 3.1 Stabilization of MFC performance

**Fig. 3:**
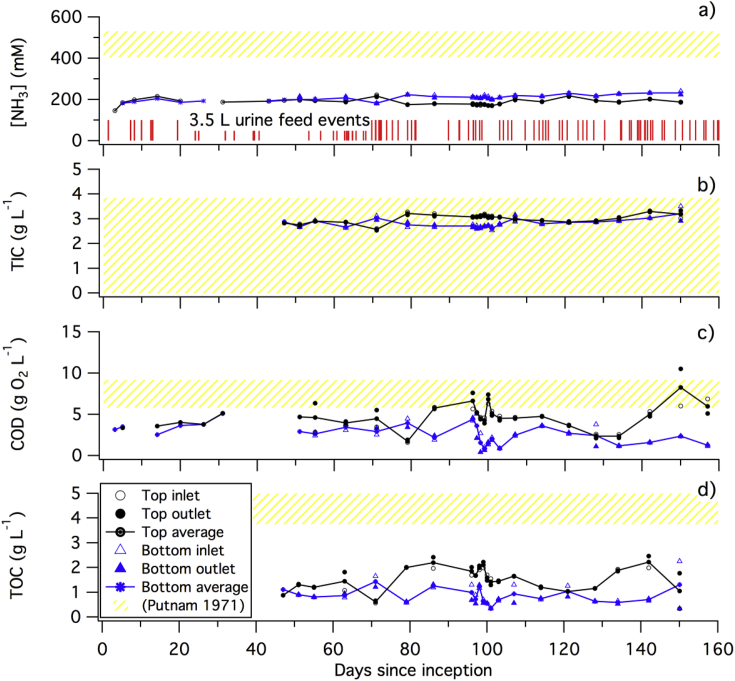
a) [NH_3_], b) TIC, c) COD, and d) TOC levels at the inlet, outlet, and averaged for each MFC stacks. The geometry of the stacks is described in section 2.1. Recorded urine feeding events are represented with vertical red bars. Range of values measured in urine samples by Putnam *et al.* are reproduced in a yellow pattern. [NH_3_] pattern is based of Total Kjehdal Nitrogen (TKN). (For interpretation of the references to color in this figure legend, the reader is referred to the Web version of this article.)

The electricity produced from two separated MFC stacks described in section 2.1 (version B) connected in series was monitored continuously over the course of 160 days (Fig. 2). Input and output [NH_3_], TIC, COD, and TOC levels for both stacks were measured on a regular basis during the same period of time (Fig. 3).

After the initial feeding and inoculation period of 24 days (Fig. S2) described earlier, voltage across the potentiometer for each stack remained between 280 mV and 350 mV (Fig. 2) with increases of 30 ± 10 mV appearing soon after some feeding events. This is in contrast to the rapid increase in cell voltage of approximately 100 mV (Fig. S2), observed soon after a feeding event during the first seven days of the inoculation process. The lowering of the potential rise over the course of the 160 days of testing can be explained by the stabilization of the biological community of the MFC stacks as observed with various MFC systems in the literature [20].

This stabilization is also observed relative to some of the chemical parameters measured, particularly [NH_3_] and TIC (Fig. 3 a and b): after the initial inoculation period, [NH_3_] stabilizes at 210 ± 20 mM with limited variation between the two stacks. This concentration is nearly half of the molar equivalent of Total Kjeldahl Nitrogen (TKN) present in urine. After 80 days, [NH_3_] in the bottom stack was found to be 40 mM higher than that in the top stack. This difference in [NH_3_] is probably due to two factors: the evaporation of NH_3_ happening in both stacks not necessarily at the same rate (the top stack is open to air while the bottom stack partially sealed under the top stack, see Fig. 1b) and the slow hydrolysis of urea that occurs preferentially in the bottom stack after a longer retention time [21]. The TIC in the top stack was slightly higher (0.2 ± 0.1 g C L^-1^ = 22 mM C) than in the bottom stack. This was most likely due to the formation of HCO_3_^−^ from urea hydrolysis, which also partially accounted for the 40 mM increase in [NH_3_] between top and bottom stacks.

Contrary to the stability of [NH_3_], the variability in COD levels over the 160-day period was more pronounced (Fig. 3, c): in the top stack the COD level was higher than in the bottom stack by 2 g O_2_ L^−1^ to 5 g O_2_ L^−1^, on average. This indicated active mechanisms for COD removal linked with the presence of microbial communities that were feeding on organic compounds from urine. Furthermore, the difference between inlet and outlet values of the top stack of 2.4 ± 1.2 g O_2_ L^−1^ (*e.g.*, days 55, 71, 96, 150 and 157) and less than 1 g O_2_ L^−1^ for the bottom stack indicates that the COD removal activity in the top stack is not as uniform as in the bottom stack. Finally, the difference in COD value between the outlet of the top stack and the inlet of the bottom stack is due to the fact that despite being connected hydraulically in series, the two stacks did not share fluids continuously: the overflow of the top stack drops to the bottom stack only when it was filled, which occurred after a urine feed event as recorded on Fig. 3, a). The rise in COD values near 140 days could be explained by a more frequent number of feeding events or a decrease in microbial activity in the top stack, the latter being the least probable because the voltage of the top stack did not drop (Fig. 2).

The variation in TOC values follows closely the same pattern as the COD values with the difference between inlet and outlet values of the top and bottom stacks of 0.75 ± 0.25 g C L^-1^ on different days, indicating a non-uniform oxidation of organic matter across both stacks. TOC levels were significantly higher in the top stack than in the bottom stack for the majority of the samples except for two sampling days: day 71 and day 150. The drop in the TOC value measured on these two days did not seem to be part of a trend and might be due to sampling error. The sustained difference between TOC levels from top to bottom stacks indicates a relatively high mineralization process and is consistent with microbial respiration.

**Fig. 4:**
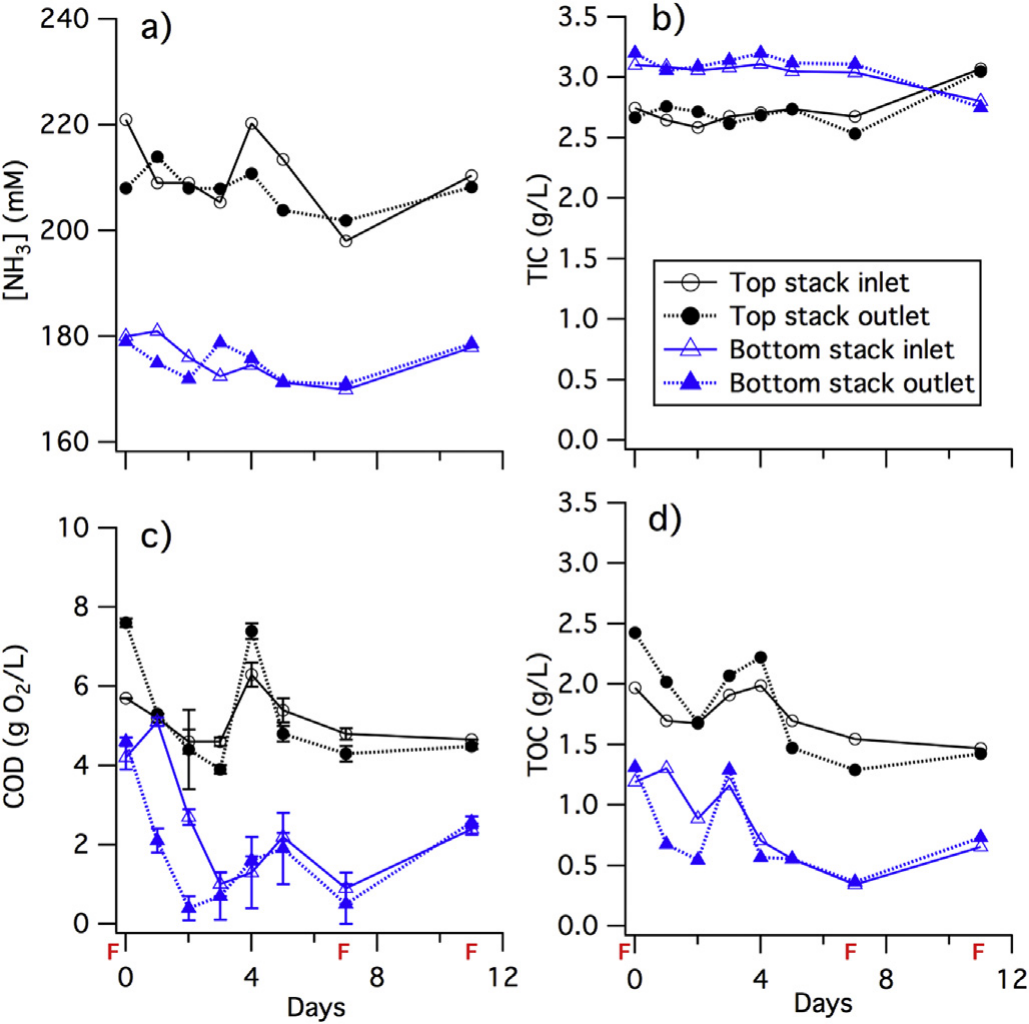
Evolution of chemical parameters a) [NH_3_], b) TIC, c) COD, and e) TOC, over the course of 12 days with three distinctive “feeding events” (marked “F”) in which 3.5 ± 0.25 L of fresh urine entered from the top MFC stack inlet.

The 12-day controlled feeding test, performed between day 96 and 107 (Fig. 4) with frequent chemical monitoring of the same parameters as previously cited, confirmed the limited impact of a single feeding event on [NH_3_] and TIC (Fig. 4 a and b): [NH_3_] remained at 210 ± 10 mM at the outlet of the top stack and 175 ± 5 mM in the bottom stack over the 12-day test period with moderate changes due to feeding events. The highest variation in [NH_3_] occurred at the inlet port of the top stack: [NH_3_] increased after 4 days of fasting to 220 mM and then decreased to 200 mM between day 4 and 7 of the test. This pattern reappeared in a similar fashion after the second feeding. This slight jump in [NH_3_] was probably due to hydrolysis of urea present in a higher concentration at the inlet (when fresh urine entered the system) than at the outlet with slight changes (±0.1 g C L^-1^) of similar pattern in TIC concentration in the top stack. This increase in [NH_3_] and TIC levelled off across the entire stack through diffusion.

Contrary to [NH_3_] and TIC, the difference in COD and TOC levels between feeding events (Fig. 4 c) and among top and bottom stacks is more consequential: all the inlet and outlet values showed a decrease short after the feeding followed by an increase a few days later. The highest drop in COD levels was observed at the outlet of the stacks: during the first 3 days after feeding, the COD level at the outlet of the top stack decreased by 50% from 8 g O_2_ L^−1^ and less than 20% at the inlet of the same stack. An even more drastic decrease of close to 90% from 4.2 g O_2_ L^−1^ occurs at the outlet and the inlet of the bottom stack. After reaching their respective minima, the COD levels increased by 4 g O_2_ L^−1^ for the top stack outlet and by 2 g O_2_ L^−1^ for the other sampling ports. TOC values followed the same trend but with less drastic changes. The increase in COD and TOC levels followed by a decrease is typical of microbial oxidation of organic waste as observed in wastewater treatment batch processes [22]. Thus, the oxidation of organics by the microbial community appeared to be the key driver for electricity production in both MFC stacks.

The differences in the microbial community on the anodes and in the anolyte were determined qualitatively by fluorescence microscopy (Fig. 6) and quantitatively by 16S rRNA metagenomic sequencing and sorting into operational taxonomic units (OTUs, Fig. 7). Fluorescence microscopy pictures of the anode felt show a large amount of dead and live microorganisms (Fig. 6a), with live microorganisms preferentially arranged along the fibers (Fig. 6b) in groups. Dead microorganisms are also present in similar areas, thus showing an active microbial biota on the anodes. The metagenomics analysis of the biota on the anodes and in the unfiltered anolyte showed different OTUs in the anolyte of the two MFC stacks as well as within the same anode (Fig. 7).

**Fig. 5:**
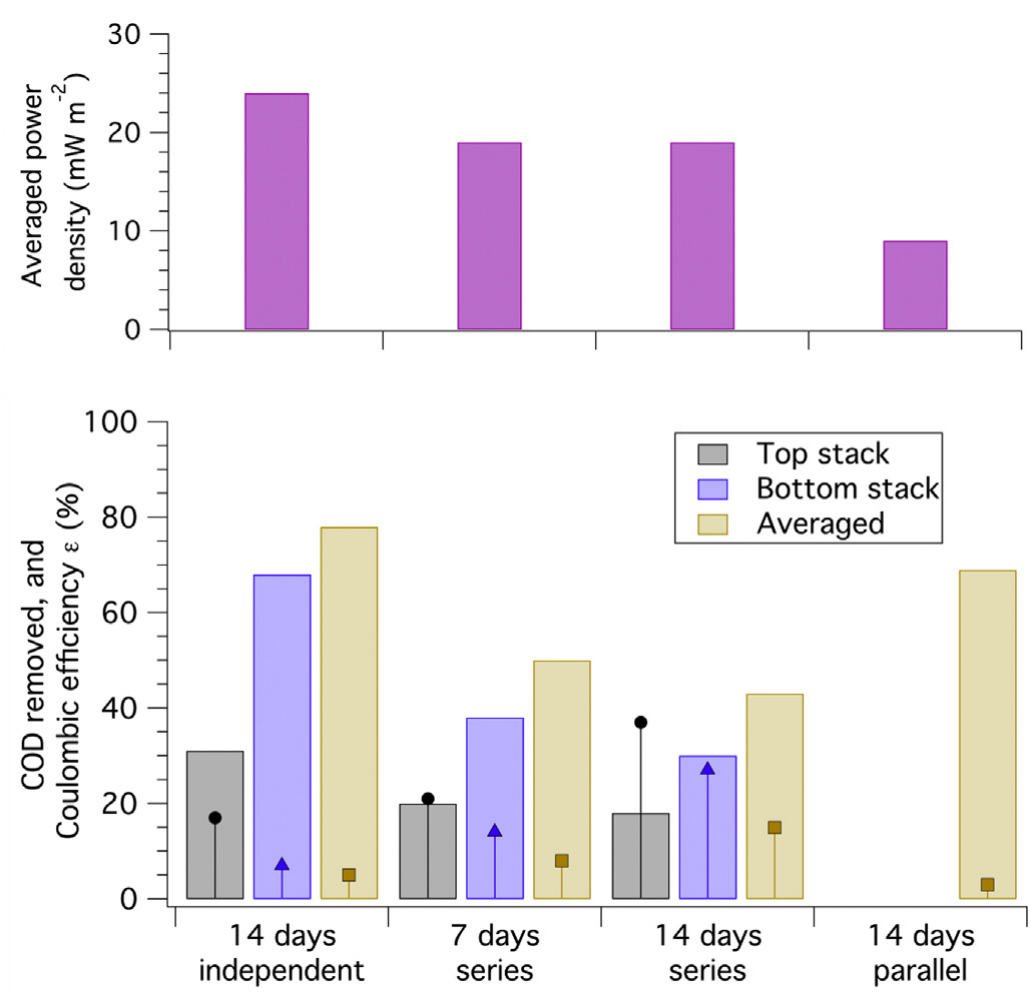
Top: specific power density (top, mW m^2^) and bottom: COD removal afficiency (bars, %) and Coulombic efficiency *ε* (sticks and markers, %) for different electrical configurations of the MFC stacks (version A): independent for 14 days (R = 12.5 Ω), in series for 7 and 14 days (R = 12.5 Ω), and in parallel for 14 days (R = 25 Ω).

**Fig. 6:**
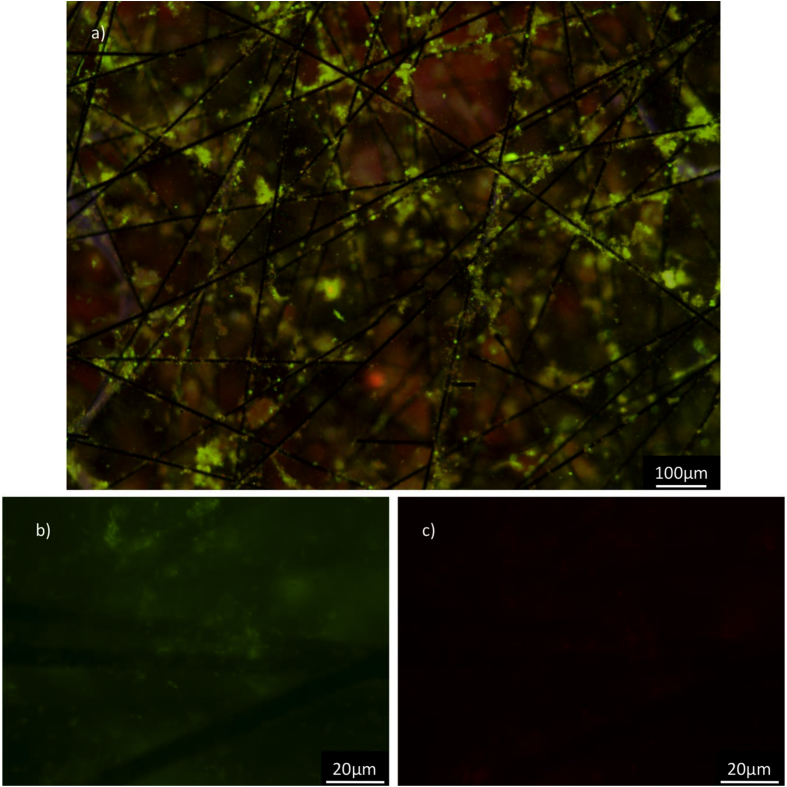
Middle section of a stained anode (see Materials and methods section) revealed under fluoresence microscopy after several months of operation in the top MFC stack: a) FITC and RHOD channels combines, b) FITC and c) RHOD channels at higher magnification with same contrast and brightness.

**Fig. 7:**
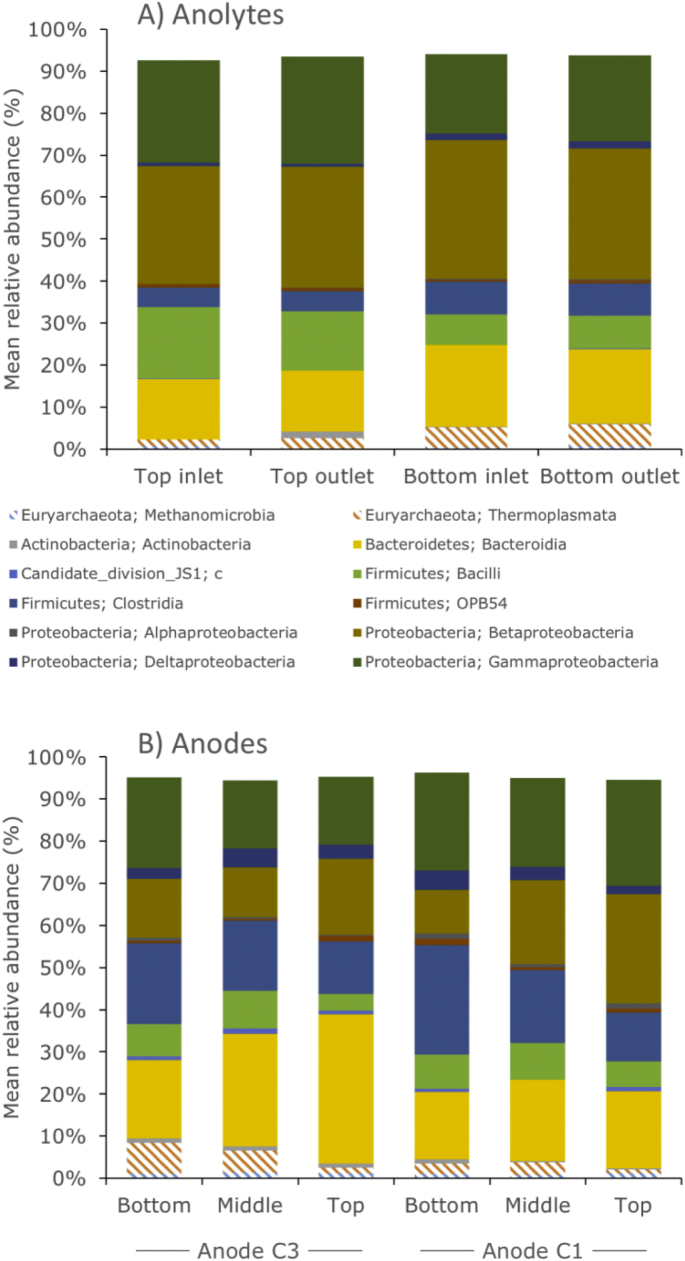
Proportion of archeal (lines) and bacterial (plain) population assigned by 16S rDNA taxonomy analysis into operational taxonomic units (OTU). Only OTUs with a minimum of 1% occurrence for any sample are represented. See Supporting Information for details on the DNA extraction method.

There were no significant differences between the composition of the OTUs at the outlet and the inlet of a specific MFC stack except for the following groups: *Archea* and *Clostridia* units were present at a higher levels in the anolyte of the bottom stack than in the top, whereas *Gamma Proteobacteria* and *Bacili* units were found at higher levels in the anolyte of the top stack than in the bottom. These differences in population might have been due to an adaptation of the biota in suspension to the different types of nutrient mixtures entering the stacks, since the top stack inlet received urine while the bottom stack inlet received the anolyte (spent urine) from the top stack outlet. Furthermore, the sampled anodes from the top stack had large differences in distribution of OTUs whether they were sampled close to the inlet (C1) or close to the outlet (C3, Fig. 1c) or whether they were sampled at the bottom, middle, or top of the anode along its vertical axis: for instance, bottom samples for C1 and C3 had the lowest *Bacteroidia* percentage and the highest *Clostridia* percentage of their respective anode, while bottom and middle parts of C1 and C3 had higher *Bacili* levels than the top of their respective anode.

Comparing the anodes and the anolyte showed no single emergent microorganism that could exclusively be linked to the electron-transfer mechanisms between bacteria and electrode. Nevertheless, the amounts of *Gammaproteobacteria* and *Clostridia* OTUs on the anodes higher than in the top anolyte could be linked to those mechanisms. Furthermore, the observed differences in microbial populations revealed an adaptation of the microbial community to its location along the vertical axis and that adaptation was probably due to a non-uniformity of the nutrient concentration, dissolved oxygen concentration, and current density along or close to the anode. A deeper understanding of the microbial communities and their role in organics oxidation in a urine medium could potentially lead to greater energy production and COD removal.

### 3.2 Energy recovery to electricity

Three electrical configurations for MFC stacks (version A) were tested for optimal electricity generation and COD removal: an independent configuration in which each stack was connected to a potentiometer of resistance R = 12.5 Ω for 14 days, a series configuration in which both stacks were connected in series with a potentiometer of resistance R = 25 Ω for two periods of 7 days each, and a parallel configuration in which both stacks were connected in parallel to a potentiometer of resistance R = 12.5 Ω for 14 days. The external resistance values should have been different for the three electrical configurations, and this was due to practical limitations and connection error. It may, nonetheless help to explain the variation in the observed behavior of the two stacks.

The highest electrical power density averaging 24 mW m^−2^ for each stack was produced when the two stacks are electrically separated (Fig. 5, top graph). This value was within range of what has been found in the literature for similar configuration [23,24]. In this case, there was no potential or current limitation that occurred when stacks were connected in series (19 mW m^−2^) or parallel (9 mW m^−2^) configurations. The overall COD removal obtained with the stacks connected independently or in series (Fig. 5, bottom graph) had the following pattern: the COD removal in the top stack was around 25 ± 5% irrespectively of the electrical configuration while the COD removal in the bottom stack was of 70% when the stacks were independent and 35% when they were in series. Moreover, the overall COD removal of the MFC system was 80% when the stacks were independent, 70% when the stacks were in parallel, and less than 50% when the stacks were connected in series.

Combined with power density measurements, Coulombic Efficiency *ε* for COD removal was dependent on electrical configuration of the stacks: the lowest overall *ε* was obtained when the stacks were connected in parallel (*ε* ≈ 3%) while the maximum overall *ε* was obtained when the stacks were connected in series (*ε* ≈ 15%). These results are in agreement with those of Oliot *et al.* on smaller scale systems [25]; they concluded that configuring MFC stacks in series was a good compromise between COD removal, power density, and Coulombic Efficiency. In addition, connecting the two stacks in series allowed for a higher output voltage and a more effective usage of the electrical energy by minimizing conversion losses for voltage ramp-up to charge a battery.

Had the stacks been connected to the correct resistance values, then the power output of the second and third configurations (series and parallel) would have been roughly double that of the individual stacks when running independently; the series connection would have produced the same current but double the voltage, and the parallel connection would have produced the same voltage but double the current. Both parameters have been shown to affect COD, efficiency, and even killing efficacy of MFCs, when studied from a principal component analysis (PCA) perspective [26]. This could have been a key factor in changing the MFC behavior and will form part of our near-term experiments to corroborate.

### 3.3 Bacterial cross-over

Catholytes were monitored for bacterial cross-over from the anolyte that contains urine and activated sludge to the catholyte compartments of the cells. Table 1 summarizes the occurrence of four common indicator microorganisms: *E. Coli*, *Total Coliform*, *Fecal Coliform*, and *Enterococcus* in selected catholytes and anolytes in four electrical configurations of the stacks as follows: 1) at open circuit for 7 days, 2) independently connected to a load, 3) connected in series, 4) or in parallel to the same load. The measured voltage at each stack for each configuration is also included. In all electrical configurations, the levels of detected microorganisms in the anolyte were too numerous to count in most instances. This is consistent with the fact that bacteria derived from activated sludge should be present in the anolyte at all time.

**Table 1:** Bacterial count (CFU per ml) of *Total Coliform*, *Fecal Coliform*, *E. Coli*, and *Enterococcus* indicator microogranisms for different electrical configurations of the stacks in the following sequence: open circuit (7 days), independent (7 days), series (14 days), and parallel (14 days). The measured voltage of each stack for each configuration is written in parenthesis in italic.

Configuration & sample type	*Total coliform*	*Fecal coliform*	*E. Coli*	*Enterococcus*
Urine	n.d.^a^	n.d.	n.d.	n.d.
Open circuit	(*top stack*: *0.62 *± *0.*02 V, *bottom stack*: *0.52 *± *0.*01 V)			
Top stack in	TNTC^b^	TNTC	TNTC	TNTC
Top stack out	TNTC	TNTC	TNTC	TNTC
Bottom stack out	TNTC	TNTC	TNTC	TNTC
Catholyte 1	22	n.d.	6.0	39
Catholyte 2	12	4.0	5.0	29
Catholyte 3	10	1.0	2.0	3.4
Independent	(*top stack: 0.45 *± *0.*04 V, *bottom stack*: *0.3 *± *0.*02 V)			
Top stack in	TNTC	n.d.	TNTC	TNTC
Top stack out	TNTC	n.d.	TNTC	TNTC
Bottom stack out	TNTC	TNTC	TNTC	8.0
Catholyte 1	>1.0	n.d.	1.0	n.d.
Catholyte 2	TNTC	n.d.	n.d.	n.d.
Catholyte 3	TNTC	n.d.	37	n.d.
Series	(*top stack*: *0.40 *± *0.*02 V, *bottom stack*: *0.24 *± *0.*01 V)			
Top stack in	6.0	n.d.	n.d.	TNTC
Top stack out	n.d.	n.d.	n.d.	TNTC
Bottom stack out	TNTC	n.d.	n.d.	n.d.
Catholyte 1	n.d.	n.d.	n.d.	n.d.
Catholyte 2	TMTC	n.d.	n.d.	n.d.
Catholyte 3	n.d.	n.d.	n.d.	n.d.
Parallel	(*top and bottom stacks*: *0.34 *± *0.*04 V)			
Top stack in	TNTC	TNTC	TNTC	TNTC
Top stack out	TNTC	TNTC	TNTC	n.d.
Bottom stack out	TNTC	n.d.	n.d.	n.d.
Catholyte 1	0	n.d.	n.d.	n.d.
Catholyte 2	TNTC	n.d.	n.d.	n.d.
Catholyte 3	3.0	–	–	–

a Non detected.

b Too numerous to count.

All indicator microorganisms were detected in all three catholyte samples when the MFC stacks were at open circuit for 7 days. When the stacks were electrically connected in series or in parallel for 14 days, only *Total Coliform* tests appeared positive while the other indicator organisms were not detected. The independent connection of the stacks for 7 days showed mix results with *Enterococcus* and *Fecal Coliform* organisms below detection limits; *E. Coli.* bacteria at or above 1 CFU mL^−1^ in two out of three catholytes. *Total Coliform* tests appeared positive in all instances.

Since no catholyte is intentionally placed in contact with the activated sludge and urine mixture of the anolyte compartment, bacterial diffusion through the terracotta membrane is the probable reason why all the indicator microorganisms are detected in the catholyte compartment. Furthermore, for stacks at open circuit, there is no electrical potential gradient between anode and cathode and diffusion through the terracotta occurs because of drying on the cathode (open to air).

When anodes and cathodes are connected to a load, the cathodic reduction of oxygen to water (eqn (2)) increases the pH in the catholyte [27] observed in this type of MFCs [28], making the catholyte less favorable for bacteria to grow [29].

(2) O_2_ + 4 e^−^ + 4 H^+^ → 2 H_2_O

There is no significant difference between the electrochemical potentials at each electrode whether the stacks are electrically connected independently or in series to the same load: the difference in concentration of indicator organisms in the catholytes between independent connection and parallel or series might be simply due to a longer period of time at which the system was run with oxygen reduction at the cathode.

The minimal bacterial cross-over during operation could imply that water present in the catholyte compartment could be used beneficially; however, the high level of *Total Coliform* bacteria indicate that the catholyte water may contain pathogens. Direct contact usage would not be recommended but indirect usage such as heavy metals precipitation could be applied [30]. The low bacterial-load reduction of the anolyte makes the presence of a post-treatment option such as electrochemical oxidation compulsory.

## 4 Conclusions

• Continuous averaged power density of 23 mW m^-2^ at a current density of 65 mA m^−2^ was produced for more than 120 days.
• COD and TOC removal was observed concomitantly with power production via anodic oxidation.
• Bacterial cross-over between anolyte and catholyte was observed at open circuit, but fewer micro-organisms are detected when MFC stacks are electrically connected in series or parallel.
• A large diversity of microorganisms was observed on the anodes and in solution; however, electricity production could not be linked to a single genus.
