# Supplementary material for: Urine microbial fuel cells in a semi-controlled environment for onsite urine pre-treatment and electricity production
Source: J Power Sources. 2018 Oct 1;400:441–8. doi: 10.1016/j.jpowsour.2018.08.051 (PMC6472131; doi:10.1016/j.jpowsour.2018.08.051)
Supplement: Multimedia component 1 [file mmc1.docx]

**Supporting information**

**Urine microbial fuel cells in a semi-controlled environment for onsite urine pre-treatment and electricity production**

Clement A. Cid^1^, Andrew Stinchcombe^2^, Ioannis Ieropoulos^2^, and Michael R. Hoffmann^1^

^1^Linde-Robinson Laboratories, California Institute of Technology, Pasadena, CA

^2^Bristol BioEnergy Centre, Bristol Robotics Laboratory, University of the West of England, BS16 1QY, UK

Number of pages: 4 (including the cover page)

Number of figures: 2

Number of tables: 0


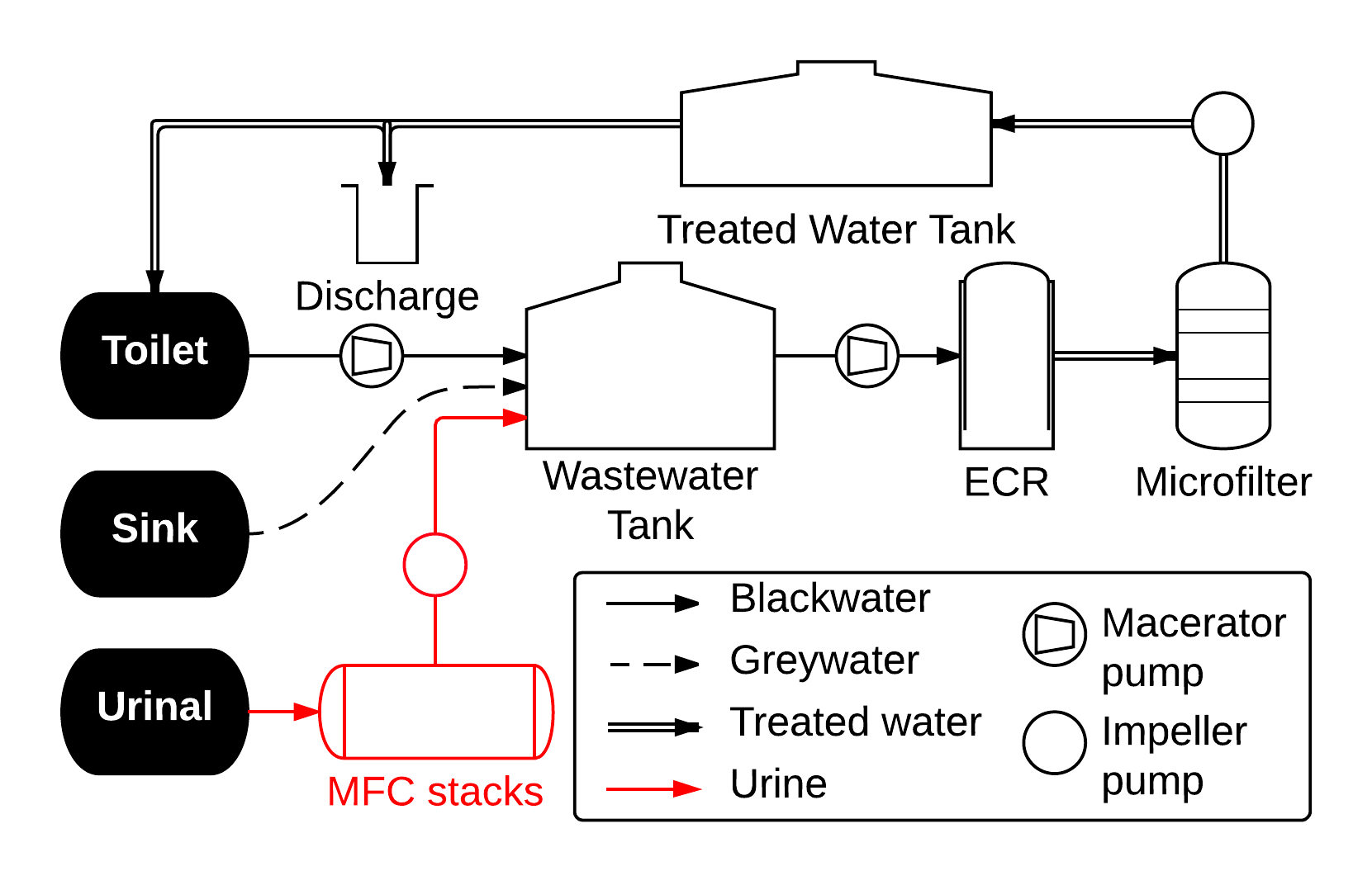


#### Figure S1: Integration of the MFC stacks within the treatment scheme of the self-contained wastewater treatment and recycling system developed by Hoffmann *et al.* [[1](#_ENREF_1)].


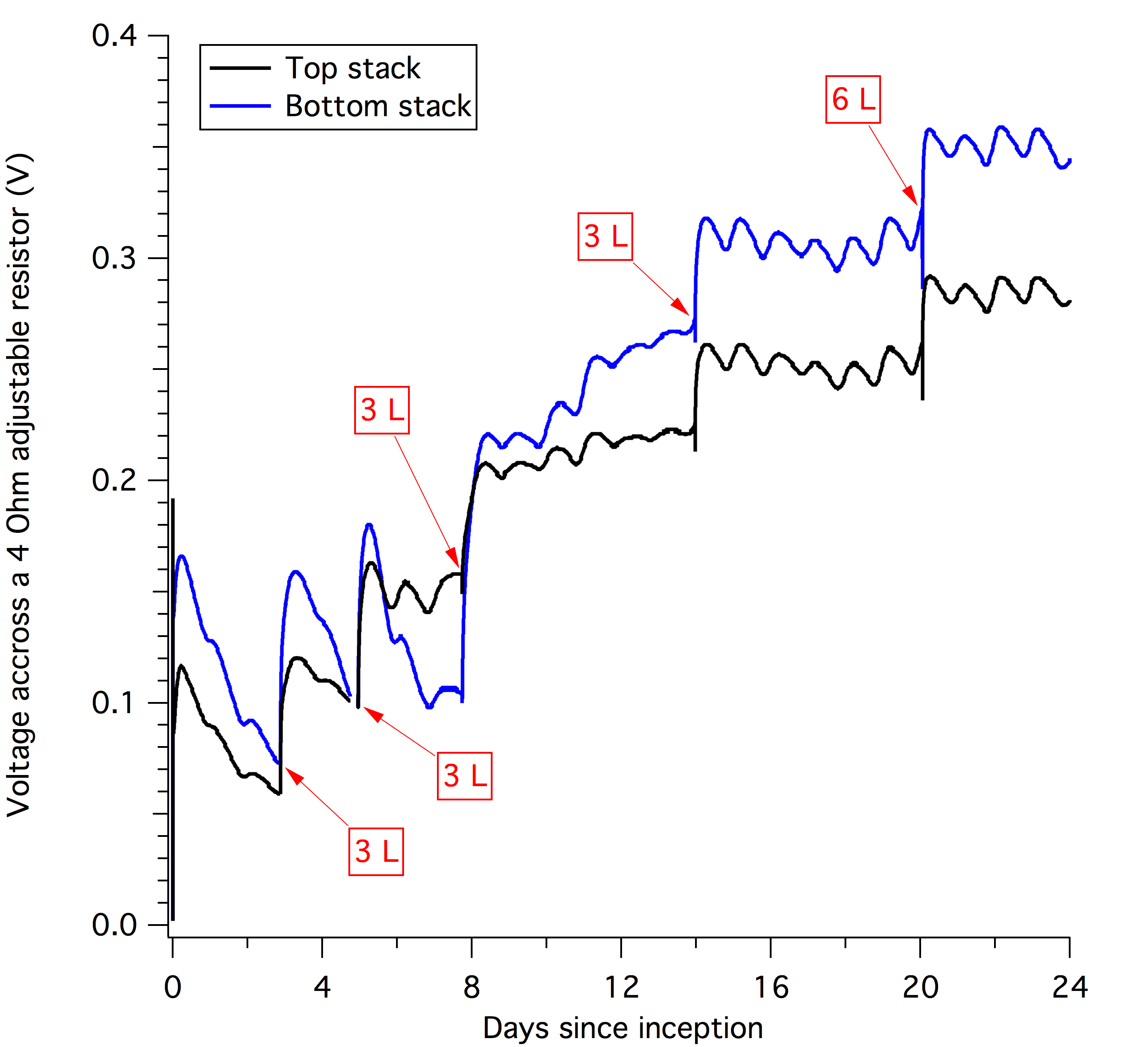


#### Figure S2: Potential measured across a 4 Ω resistor for each independent stack. The red arrows indicate a feeding event: each stack was slowly drained of the anolyte volume written and replaced by the same quantity of fresh urine.

**Genomic DNA extraction protocol (based on Mo Bio and modified by the Orphan research group at the California Institute of Technology):**

Anode felts were cut in pieces of 0.5 cm by 1 cm. Each felt was placed in a 15-mL conical centrifuge tube with 10 mL of a 70% ethanol solution in an ice bath. The mixture was sonicated for three sessions of 10 s each at 5 W power with 30 s break between sessions. After sonication, the felt was discarded and the remaining mixture was quickly filtered using a disposable filter funnel system with 0.45 µm filter membrane. Anolyte samples were directly filtered on a similar disposable filter funnel.

After filtration, the filter membrane was carefully inserted in the 5-mL Mo Bio PowerWater® Bead Tube with the top (cell) side of the membrane facing inwards. After adding 1 mL of Mo Bio PowerWater® PW 1 solution at 65 ºC, the tube was briefly vortexed and incubated at 65 ºC for 10 min in a heat block. At the end of the incubation period, the tube was attached vertically using Mo Bio Vortex Adapter and vortexed at maximum speed for 5 min. The tube and its content were then centrifuged at 4,000•*g* force for 1 min so all the supernatant could be transferred to an autoclaved 2-mL centrifuge tube and centrifuged at 13,000•*g* force for 1 min. The rest of the protocol was identical to steps 11 through 24 of the Experienced User Protocol published by Mo Bio [[2](#_ENREF_2)].

**16S rRNA gene sequencing and processing from Case *et al.* [**[**3**](#_ENREF_3)**]:**

Preparation for sequencing of the V4 region of the 16S rRNA gene was performed with universal primers according to the protocol recommended by the Earth Microbiome Project (http://www.earthmicrobiome.org/emp-standard-protocols/16s/) [[4](#_ENREF_4), [5](#_ENREF_5)], with minor modifications described elsewhere [[6](#_ENREF_6)]. Raw sequences were generated on an Illumina MiSeq platform at Laragen, Inc. (Los Angeles, CA). In-house data processing was completed in QIIME1.8.0 and included joining paired ends, quality trimming, chimera checking, 97% OTU clustering, singleton removal, PCR contaminant removal, 0.01% relative abundance threshold removal, and rarefaction to 16,051 sequences per sample. Taxonomic assignments were generated according to an appended version of the Silva 115 database (for details, see [[6](#_ENREF_6)]).

**References:**

1. Hoffmann, M.R., et al., *Self-contained, pv-powered domestic toilet and wastewater treatment system*. 2013, Google Patents.

2. Mo Bio. *PowerWater® DNA Isolation Kit Sample*. 2015; Available from: <https://mobio.com/media/wysiwyg/pdfs/protocols/14900-S.pdf>.

3. Case, D.H., et al., *Methane seep carbonates host distinct, diverse, and dynamic microbial assemblages.* MBio, 2015. **6**(6): p. e01348-15.

4. Caporaso, J.G., et al., *Ultra-high-throughput microbial community analysis on the Illumina HiSeq and MiSeq platforms.* The ISME journal, 2012. **6**(8): p. 1621-1624.

5. Caporaso, J.G., et al., *Global patterns of 16S rRNA diversity at a depth of millions of sequences per sample.* Proceedings of the National Academy of Sciences, 2011. **108**(Supplement 1): p. 4516-4522.

6. Mason, O.U., et al., *Comparison of archaeal and bacterial diversity in methane seep carbonate nodules and host sediments, Eel River Basin and Hydrate Ridge, USA.* Microbial ecology, 2015. **70**(3): p. 766-784.
